# Artificial intelligence and oral photography: an approach to the epidemiology of dental caries

**DOI:** 10.11606/s1518-8787.2025059006910

**Published:** 2026-01-12

**Authors:** Luiz Roberto Augusto Noro, Maria Cristina Manzanares Céspedes

**Affiliations:** I Universidade Federal do Rio Grande do Norte. Departamento de Odontologia. Programa de Pós-graduação em Saúde Coletiva. Natal, RN, Brasil; II Universidad de Barcelona. Facultat de Medicina i Ciències de la Salut. Departament de Patologia i Terapèutica Experimental. Barcelona, Spain

**Keywords:** Dental Caries, Artificial Intelligence, Epidemiology, Diagnosis, Dental Photography

## Abstract

**OBJECTIVE:**

Dental caries is an important public health issue due to its high prevalence around the world, its impact on people’s quality of life, and the existence of effective methods to control and prevent it. This study aims to find the state of the art regarding the diagnosis of caries with the use of artificial intelligence in the scientific literature, which could represent future advances in its use in oral health epidemiology.

**METHODS:**

A scoping review was carried out based on a search strategy on the main health databases that found 1,439 articles by descriptors or keywords related to caries, diagnosis, and artificial intelligence.

**RESULTS:**

After analysis, 17 scientific articles composed the final sample. Of these, 94.1% are quite recent, published from 2020 onward. Although the articles were based on a clinical perspective, their objectives, results, and conclusions signal the possible effectiveness of artificial intelligence as a strategic tool for epidemiology. Frontal, lateral, and occlusal photographs served to diagnose caries on all sides of the teeth.

**CONCLUSION:**

It is essential to invest in alternatives related to artificial intelligence and oral photography to replace traditional epidemiological surveys, which would enable the full development of oral health surveillance.

## INTRODUCTION

Dental caries is an important public health issue^1^ since its average prevalence reaches 29% of the global population^2^. Its aggravation leads to pain, significantly impacting individuals’ quality of life and absenteeism at work^3,4^. Nevertheless, governments can intervene to prevent caries with effective and efficacious collective measures, such as the fluoridation of the public water supply^5^.

The approach to caries in the Brazilian Unified Health System still characterize an enormous challenge, especially due to the accumulation of the Brazilian population’s needs and the organization of local planning that focuses on equity and follows an epidemiological reference^6^.

Epidemiological surveys in Brazil^7^can find the national magnitude of the problem but they are unable to identify the specific oral health needs of a population group in a territory. Moreover, finding families and individuals at risk in a community requires applying observation instruments to the entire population group rather than only to a sample, rendering traditional epidemiological surveys to assess the local situation of dental caries unfeasible.

Thus, artificial intelligence offers an alternative to detect caries lesions, especially from images. Studies have used dental radiography^10,11^for such diagnosis, which would avoid the need for clinical examinations. However, this alternative is unfeasible for epidemiological purposes due to its high cost for public health systems and the risk related to ionizing radiation^12^.

Considering this difficulty, studies indicate the possibility of detecting caries lesions from photographic images and artificial intelligence, which can contribute to an epidemiological reference^13,14^ that is indispensable for local oral health planning.

Thus, this study aims to synthesize scientific evidence related to the diagnosis of caries via photographic images and artificial intelligence that could represent perspectives of its use in oral epidemiology.

## METHODS

### Type of Study

This scoping review^14^ was proposed to map the scientific production in health that would evince the advances and challenges related to diagnosing caries with images and artificial intelligence. The recommendations proposed by the Joanna Briggs Institute^15^ and its results were adapted for this review according to the Preferred Reporting Items for Systematic Review and Meta-Analyses Extension for Scoping Reviews^16^.

To build the initial question of this research, the Population/Concept/Context strategy was used, in which Population equaled people with caries; Concept, epidemiological surveys of caries by imaging; and Context, use of artificial intelligence. These elements built the guiding question of this research: “What is the current situation of epidemiological caries surveys that are developed by images using artificial intelligence?”

However, a search strategy on BVS: *Biblioteca Virtual de Saúde* (Virtual Health Library), Medical Literature Analysis and Retrieval System Online (MEDLINE), Web of Science (WoS), and Abstract and Citation Database of Peer-Reviewed Literature (Scopus) found no articles that met those criteria.

Due to this inadequacy, the PCC strategy was reformulated, in which the Concept was defined as caries diagnosis by imaging and the original Population (people with caries) and Context (use of artificial intelligence) criteria were retained as in the first search. This change obtained the following guiding question: “What is the current situation of caries diagnosis developed by images and artificial intelligence?”

### Information Sources and Search Strategy

The searches were carried out from May to August 2024, with a publication deadline of August 20, 2024, on BVS, MEDLINE, WoS, and Scopus. Based on the relevant terms on Medical Subject Headings, searches were carried out according to the specificities of each database. Such strategy is detailed in Chart 1.

**Chart 1:** Search strategy used in databases based on descriptors. CAPES Journals, Brazil, 2024.

Database	Search strategy
BVS	((artificial intelligence or machine learning or deep learning or fuzzy logic or computational intelligence or machine intelligence or computer reasoning or algoritm)) AND ((caries or lesion or dental cavity or dental cavities or white spot or decay)) AND ((diagnostic or diagnosis or detection or detecting or classification or classifying or screening or prediction or predicting)) Title, abstract, subject
MEDLINE	(“artificial intelligence”[Title] OR “machine learning”[Title] OR “deep learning”[Title] OR “fuzzy logic”[Title] OR “computational intelligence”[Title] OR “machine intelligence”[Title] OR ((“computability”[All Fields] OR “computable”[All Fields] OR “computating”[All Fields] OR “computation”[All Fields] OR “computational”[All Fields] OR “computations”[All Fields] OR “compute”[All Fields] OR “computed”[All Fields] OR “computer s”[All Fields] OR “computers”[MeSH Terms] OR “computers”[All Fields] OR “computer”[All Fields] OR “computes”[All Fields] OR “computing”[All Fields] OR “computional”[All Fields]) AND “reasoning”[Title]) OR “algorithm”[Title] AND (“caries”[Title] OR “lesion”[Title] OR “dental cavity”[Title] OR “dental cavities”[Title] OR “white spot”[Title] OR “decay”[Title]) AND (“diagnostic”[Title] OR “diagnosis”[Title] OR “detection”[Title] OR “detecting”[Title] OR “classification”[Title] OR “classifying”[Title] OR “screening”[Title] OR “prediction”[Title] OR “predicting”[Title])
WoS	(artificial intelligence or machine learning or deep learning or fuzzy logic or computational intelligence or machine intelligence or computer reasoning or algoritm) AND (caries or lesion or dental cavity or dental cavities or white spot or decay) AND (diagnostic or diagnosis or detection or detecting or classification or classifying or screening or prediction or predicting) (Title)
Scopus	TITLE-ABS-KEY (artificial AND intelligence OR machine AND learning OR deep AND learning OR computational AND intelligence OR algorithm) AND TITLE-ABS-KEY (caries OR lesion OR dental AND cavity OR dental AND cavities) AND TITLE-ABS-KEY (diagnostic OR diagnosis OR detection OR detecting OR classification OR classifying OR screening OR prediction OR predicting)

CAPES: *Coordenação de Aperfeiçoamento de Pessoal de Nível Superior*; BVS: *Biblioteca Virtual de Saúde* (Virtual Health Library); MEDLINE: Medical Literature Analysis and Retrievel System Online; WoS: Web of Science; Scopus: Abstract and Citation Database of Peer-Reviewed Literature.

The Systematic Review and Meta-Analyses Extension for Scoping Reviews flowchart with the investigative path related to article retrieval, the definition of the selection and eligibility criteria, and the evaluation and inclusion of the articles is available in Figure.

**Figure:**
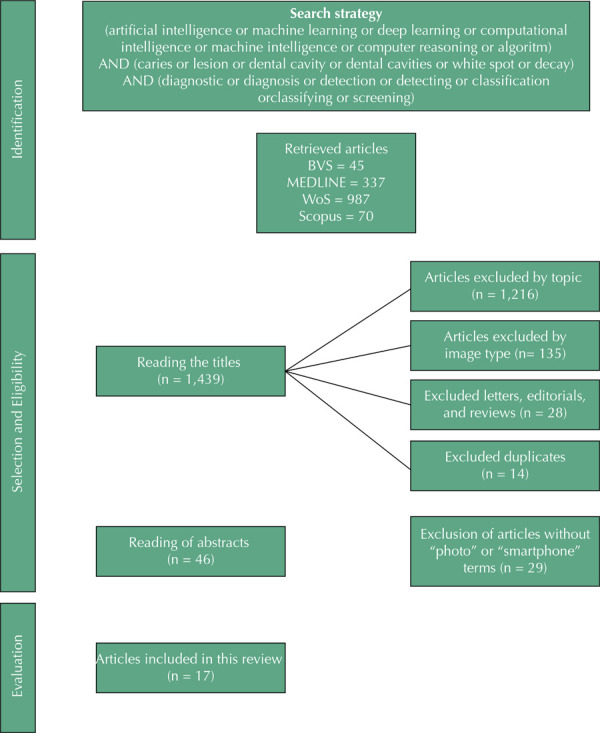
Preferred Reporting Items for Systematic Review and Meta-Analyses Extension for Scoping Reviews Flowchart.

BVS: *Biblioteca Virtual de Saúde* (Virtual Health Library); MEDLINE: Medical Literature Analysis and Retrievel System Online; WoS: Web of Science; Scopus: Abstract and Citation Database of Peer-Reviewed Literature.

Note that the gray literature was ignored to preserve the validity, reliability, and consistency of the analyses of scientific production in peer-reviewed journals.

### Data Collection

From the information in the articles on the databases, a specific instrument was structured on Microsoft Excel to tabulate the data and extract the main results.

Only complete articles published in English (with no time limitation) that contained descriptors or keywords related to caries, diagnosis, and artificial intelligence were included in this review.

### Data Analysis

The articles were independently reviewed by two reviewers, whose disagreements were resolved by consensus. First, all titles were read to find the pertinent studies for this research. If reading their title failed to define the eligibility of a study, the abstract of that scientific article was analyzed. The variables to compose this study were related to authorship, year, objectives, type of study, study population, data source, variables, main results, and conclusion.

All articles at the end of this search strategy were exported to Mendeley, a free reference manager, to organize the documents for later analysis.

## RESULTS

The initial search in the defined databases retrieved 1,439 scientific articles that were published in English. This review excluded 1,216 of them because they failed to align themselves with the theme of this research and another 135 because they were related to images such as radiography, ultrasound, infrared, among others. It also excluded editorials, letters, and reviews (28) and 14 duplicates as they failed to directly meet the focus of this research. Of the remaining 46 articles, this study excluded 29 that failed to contain the terms “photo,” “smartphone,” “artificial intelligence,” “deep learning,” or “machine learning;” strategic elements to address the possibility of articulating epidemiology with artificial intelligence based on images. Thus, 17 articles composed the sample of this scoping review.

Analyzing these articles shows that only one was published before 2020. Chart 2 lists the articles and their title, main author, and year of publication.

**Chart 2:** Title, lead author, and year of publication of the articles in this scoping review, 2024.

Article title	Author/year
The accuracy of asynchronous tele-screening for detecting dental caries in patient-captured mobile photographs: a pilot study	Qari et al.^14^ (2023)
Assessing a smartphone app (AICaries) that uses artificial intelligence to detect dental caries in children and provides interactive oral health education: protocol for a design and usability testing study	Xiao et al.^17^ (2021)
Automated caries detection with smartphone color photography using machine learning	Duong et al.^18^ (2021)
Deep learning application in dental caries detection using intraoral photographs taken by smartphones	Thanh et al.^19^ (2022)
Mobile photographic screening for dental caries in children diagnostic performance compared to unaided visual dental examination	Estai et al.^20^ (2021)
Artificial Intelligence-powered smartphone application AlCaries improves at home dental caries screening in children: moderated and unmoderated usability test	Al-Jallad et al.^21^ (2022)
Detection of dental caries in oral photographs taken by mobile phones based on the YOLOv3 algorithm	Ding et al.^22^ (2021)
Development and evaluation of deep learning for screening dental caries from oral photographs	Zhang et al.^23^ (2020)
Visual diagnostics of dental caries through deep learning of non-standardized photographs using a hybrid yolo ensemble and transfer learning model	Tareq et al.^24^ (2023)
A computer-aided automated methodology for the detection and classification of occlusal caries from photographic color images	Berdouses et al.^25^ (2015)
Detecting white spot lesions on dental photography using deep learning: a pilot study	Askar et al.^26^ (2021)
A hybrid mask RCNN-based tool to localize dental cavities from real-time mixed photographic images	Rashid et al.^27^ (2022)
Caries detection on intraoral images using artificial intelligence	Kühnisch et al.^28^ (2022)
Caries detection with tooth surface segmentation on intraoral photographic images using deep learning	Park et al.^29^ (2022)
Simultaneous detection of dental caries and fissure sealant in intraoral photographs by deep learning: a pilot study	Xiong et al.^30^ (2024)
Automated detection of posterior restorations in permanent teeth using artificial intelligence on intraoral photographs	Engels et al.^31^ (2022)
Employing CNN ensemble models in classifying dental caries using oral photographs	AlSayyeda et al.^32^ (2023)

RCNN: Region-Based Convolutional Neural Networks; CNN: Convolutional Neural Networks.

All articles diagnosed caries by using photography to simulate the images dental surgeons observe during clinical examinations. In total, seven studies^14,17^ used smartphones to photograph participants’ mouths, three used digital cameras^23^, and seven other studies chose to take intraoral photographs using professional photographic equipment^26^.

Moreover, three studies^14,17,21^used methods that enabled parents or family members to take photographs without the need for a healthcare provider. Along the same lines, a study^20^ proposed a software to support dental surgeons and patients in the preliminary stages of diagnosis. All other studies were directly related to the possibility of dental surgeons detecting dental caries outside the clinic.

Sample units showed great variety. Some studies focused on extracted teeth, using artificial intelligence to diagnose caries based on images of these teeth. Of these, one study^24^ focused on anterior teeth, whereas two^18,25^ analyzed the occlusal aspect of premolars and molars (the most affected by dental caries). Also, two studies^17,21^ had parents and children taking photographs in their homes to provide them greater knowledge and enable family members to detect caries early due to such health education. Another study^14^ promoted self-photography with patients of a clinic in a Dentistry course also under such educational perspective. Most of the studies carried out their research with patients from teaching clinics or health services. Four of these were carried out with patients from dental clinics or hospitals, of which one study^28^ worked with photographs in patients’ medical records, whereas the other three involved photographs from patients who were receiving treatment^22,27,30^; six studies included patients from dentistry courses, two^31,32^ of which investigated photographs in the course records from research conducted for educational purposes and four that worked directly with patients^19,23,26,29^. An epidemiological study^20^ targeted children aged from four to 14 years, carrying clinical examinations and photographs at the school. All the images of the studies in this scoping review corresponded to photographs of the mouth, with special emphasis on the teeth.

Still, two articles^17,21^ ignored detailing the type of photograph they used as these qualitative studies focused on obtaining feedback on the usability of their application and flow between family members regarding their health education strategies. Moreover, eight studies used photographs of specific teeth^18,24^ and three took photographs of specific areas of the dental arch^19,23,27^. Of greater interest to this study, four studies photographed the occlusal portion of participants’ teeth, of which one^30^ used only such photographs; another^22^ added frontal photographs (buccal surface of the anterior teeth); and two^14,20^, in addition to the frontal photographs, included lateral photographs (buccal surface of the posterior teeth).

Regarding the type of study, two studies showed a qualitative design^17,21^, whereas the others, an accuracy one. Of these, one study compared the pattern of oral photographs taken by dental students and patients^14^, whereas four others compared the results of the clinical examination with the photographs^20,25,29,30^. One study^27^ associated photographs and radiographs to compare its results with a previously proposed artificial intelligence system. Nine others only compared the results of artificial intelligence system analyses of photographs^18,19,22^.

Table shows a consolidated report on the addressed aspects, and Chart 3 details the main conclusions of the analyzed articles.

**Table:** Total and percentages of the variables in this scoping review, 2024.

Variable		Total	%
Studied period	After 2020	16	94.1
	Up to 2019	1	5.9
Photographic equipment	Smartphones	7	41.2
	Intraoral camera	7	41.2
	Digital Camera	3	17.6
Study objective	Detect dental caries in non-clinical settings	13	76.5
	Analyze photographic taken by parents or relatives	3	17.6
	Develop software to support the preliminary stages of diagnosis	1	5.9
Sampling units	Patients of Dentistry courses	6	35.3
	Patients in clinics or hospitals	4	23.5
	Extracted teeth	3	17.6
	Parents and children at home	2	11.8
	Children aged from 4 to 14 years	1	5.9
	Self-photography (patients of Dentistry course)	1	5.9
Photographed mouth region	Individual teeth	8	47.1
	Occlusal areas	4	23.5
	Specific regions of the dental arch	3	17.6
	No identification of the photographed region	2	11.8
Study design	Qualitative	2	11.8
	Accuracy	15	88.2

**Chart 3:** Main conclusion of the articles in this scoping review according to lead author and year of publication, 2024.

Author/year	Conclusion
Qari et al.^14^ (2023)	The teletriage based on reviewing photographs participants took can be a valid and reliable alternative to clinical examination.
Xiao et al.^17^ (2021)	With the AlCaries application, parents can use regular smartphones to take pictures of their children’s teeth, detect early childhood caries, reduce the risk of caries, and seek treatment at an early stage of caries.
Duong et al.^18^ (2021)	Integrated artificial intelligence application to help people give an early warning about their oral health conditions and help dentists to reduce clinical examination times.
Thanh et al.^19^ (2022)	YOLOv3 and Faster RCNN are promising artificial intelligence applications to detect cavitated caries lesions but the accuracy and sensitivity of the four models in detecting early caries lesions remained lower than expected.
Estai et al.^20^ (2021)	The photographic approach to dental screening produced an acceptable diagnostic level of caries detection, particularly in children with deciduous dentition.
Al-Jallad et al.^21^ (2022)	Perceived benefits of using the AICaries app include convenient screening of caries lesions at home, caries lesion risk information, education, and family involvement.
Ding et al.^22^ (2021)	Deep learning algorithms can improve the unequal distribution of public health resources by using photographs taken from smartphones for detection (especially of primary caries lesions).
Zhang et al.^23^ (2020)	Deep learning algorithms can improve public health by automatic screenings of diseases using photographs captured from cameras, which can be used for self-examination.
Tareq et al.^24^ (2023)	The model can improve access to oral healthcare in rural areas with limited resources and may aid automated diagnostics and advanced teledentistry applications.
Berdouses et al.^25^ (2015)	Objective, fully automated caries diagnostic system for occlusal caries lesions with similar or better performance than a trained dental surgeon.
Askar et al.^26^ (2021)	Deep learning has shown satisfactory accuracy in detecting white spot lesions, particularly fluorosis.
Rashid et al.^27^ (2022)	The developed tool proved to effectively detect carious regions by dental photographs
Kühnisch et al.^28^ (2022)	It was possible to achieve more than 90% agreement in caries detection using the artificial intelligence method with standardized photographs of a single tooth.
Park et al.^29^ (2022)	Deep learning with tooth surface segmentation holds promise for detecting caries lesions in intraoral camera photographic images, with time and cost savings.
Xiong et al.^30^ (2024)	ToothNet achieved simultaneous multitasking detection in intraoral photographs, with advantages in the detection of caries lesions and equivalence in the detection of fissure sealants when compared to the clinical examination performed by the dentist.
Engels et al.^31^ (2022)	It was possible to automatically categorize types of posterior restorations in intraoral photographs with good accuracy.
AlSayyeda et al.^32^ (2023)	The proposed set structure for caries lesion detection is a step to build an automated and effective support system.

RCNN: *Region-Based Convolutional Neural Networks.*

## DISCUSSION

Although the emergence of artificial intelligence with the potential to intervene in health processes dates back more than 70 years, its limitations prevented its wide acceptance and application, which will occur with the advent of deep learning^33^. As in this study, although the first analyzed article was published in 2015, 94.1% of the sample emerged from 2020 onwards, which enables us to state that interest in the topic is quite recent.

Preliminary studies showed the large scientific production related to the clinical diagnosis of dental caries mediated by artificial intelligence (always according to the feasibility of these applications for dental clinics, under the logic of individual care). These studies reported advances in artificial intelligence, especially in predicting (planning of preventive dental care and oral hygiene care) and diagnosing caries (patients at higher risk)^34,35^. However, most of these studies were ineligible for this scoping review due to their interest in epidemiology.

Although this review retrieved no articles that directly referred to epidemiology, several studies^18,20,22,31^ evinced the need for specific research on artificial intelligence under this focus. Such indications are in line with the need to reach families and individuals at risk in communities.

Regarding the objectives of the studies in this present review, some sought to empower parents or family members for the early detection of caries and its systematic follow-up, greatly collaborating with health education actions^14,17,21,27^. They aimed towards reducing the risk of caries and treating the early stage of caries^17^, which would be essential for the effective development of oral health surveillance.

However, articles that aimed to detect dental caries based on artificial intelligence and images outside the clinical environment stood out. In view of these characteristics, a potential contribution in using these tools in activities to identify families and individuals at risk in an area covered by a primary health care unit would refer to its reduction of spontaneous demand and its increase in programmed demand under the principles of equity.

Studies with images such as periapical, interproximal, and panoramic radiographs, ultrasonography, fluorescence, or infrared would show no epidemiological feasibility due to their cost and difficulty of access; thus, this research excluded them.

Considering this limitation, this study chose articles using photographs of volunteers’ mouths to meet its objective. Photographs are quite common in dental education, especially to observe the before and after of treatments.

This type of dentistry technique to record detailed images of patients’ mouth offer oral healthcare providers a clear and accurate view of dental structures and soft tissues, aiding diagnosis and treatment planning.

However, using this type of equipment would make it unfeasible to diagnose caries by photographic images due to the cost of the equipment and the risks related to its use in certain territories. Thus, an alternative to this difficulty could refer to smartphones (seven studies) or digital cameras (three studies). While smartphone cameras may fail to offer the same image quality as their professional or intraoral counterparts, their wide availability, cost-effectiveness, and applicability in public health research make them promising tools for this purpose^35^. Moreover, artificial intelligence advances show the ability to improve the quality of the original photographic image by specific technology^36^.

Another important element refers to the type of photographic record according to the sample unit proposed by the articles. The photographs of specific teeth or areas of each dental arch were related to aspects regarding the accuracy of details and tests based on artificial intelligence, which fail to exactly correspond to the purposes of this study. However, six articles brought a fundamental contribution towards a solution for the development of epidemiological studies as they focused on areas of the mouth, and their dataset indicated an approximation of the diagnosis of caries. Studies based on frontal and lateral photographs diagnosed caries on the buccal surfaces of the teeth, whereas that of occlusal portions focused on diagnosing caries on the portions with the highest occurrence of dental caries (occlusal and proximal)^37^.

Considering the objectives in the retrieved articles, most constituted accuracy studies, which allows us to evaluate how much the results of a measurement correspond to the true state of the analyzed phenomenon^38^. The analysis showed that most studies signaled promising results in using oral photographs as substitutes for the clinical examinations in epidemiological surveys.

The conclusion of some articles offered elements that support future studies on the use of artificial intelligence in epidemiology, evincing that photographic evaluations showed similar or better performance than that in epidemiological surveys on dental surgeons’ examinations^25,26,31,32^. Reinforcing this perspective, studies have showed satisfactory results with artificial intelligence systems based on photographic images^20,26,27,32^. An article^20^ drew attention to the possibility of auxiliary personnel producing accurate photographs, which would be very important for a greater involvement of oral health teams in epidemiological surveys. Similarly, some articles^14,17,21,27^ reported very satisfactory results from parents’, other family members’, or patients’ photographs.

The conclusions in the retrieved studies that proved decisive for the contribution of this review (i.e., that these tools show potential) stem from those articles the objectives of which were related to the clinic (but which significantly contribute to solutions for collective health). Studies have signaled the possibility of improving public health by decreasing unequal distribution of resources^22^, enabling the automatic screening of suspected cases of diseases^23^, and offering access to oral health in rural areas^24^. Moreover, a study evinced potentially cost-effective and user-friendly screening^29^ to provide reliable, affordable, and less invasive, dangerous, and time-consuming dental caries lesion screening.

The analysis of the results of this study shows a positive view of the possibilities of artificial intelligence in health area, as per a study that highlighted significant benefits for advances in patient access to health services^39^.

However, despite the technological advances and added value for decision-making processes in health care provided by artificial intelligence, other challenges continue to concern patients and healthcare providers. The careful, ethical, and humanized use of these resources by healthcare providers must prioritize respect for patients, ensuring that technology configures an ally in care. Social and ethical implications, especially those related to privacy and security measures for patient data and the actual capacity for health monitoring should be part of this care^40^.

## FINAL CONSIDERATIONS

There is an urgent need to develop solutions based on scientific evidence and technological advances that allow healthcare providers (especially those in primary care) to develop local oral health planning based on an epidemiological framework.

It is possible to signal the future adoption of oral photographs as strategic instruments to replace the complex logistics and the imperative need to involve dental surgeons in epidemiological surveys (the only reference for collective diagnosis).

It is also important to note the satisfactory accuracy for epidemiological surveys in several studies that used the diagnosis of caries mediated by artificial intelligence from photographs despite lacking the ideal result for clinical practice.

Based on the evidence in this review, it would be essential to develop specific studies that could add advances in artificial intelligence to the Brazilian epidemiological reality.

And, once the advances are achieved, it would be essential to include this type of information and images in the Citizen’s Electronic Health Record e-SUS Primary Healthcare^41^, thus enabling oral health surveillance.
