# Supplementary material for: Artificial intelligence and oral photography: an approach to the epidemiology of dental caries
Source: Rev Saude Publica. 2026 Jan 12;59:e53. doi: 10.11606/s1518-8787.2025059006910 (PMC12713531; doi:10.11606/s1518-8787.2025059006910)
Supplement: Translation

# Translation: Inteligência artificial e fotografia bucal: aproximação à epidemiologia da cárie dentária

**Authors:** Luiz Roberto Augusto Noro, Maria Cristina Manzanares Céspedes

**Affiliations:** I Universidade Federal do Rio Grande do Norte. Departamento de Odontologia. Programa de Pós-graduação em Saúde Coletiva. Natal, RN, Brasil; II Universidad de Barcelona. Facultat de Medicina i Ciències de la Salut. Departament de Patologia i Terapèutica Experimental. Barcelona, España

**Keywords:** Cárie Dentária, Inteligência Artificial, Epidemiologia, Diagnóstico, Fotografia Dental

## Abstract

**OBJETIVO:**

A cárie dentária configura-se como importante problema de saúde pública, considerando sua alta prevalência ao redor do mundo, seu impacto na qualidade de vida das pessoas e a existência de métodos eficazes para seu controle e prevenção. O objetivo do presente estudo foi identificar na literatura científica o estado da arte relativo ao diagnóstico da cárie por meio da inteligência artificial que pudesse representar futuros avanços da sua utilização na epidemiologia em saúde bucal.

**MÉTODOS:**

Foi realizada revisão de escopo a partir da estratégia de busca definida nas principais bases da área da saúde, nas quais foram identificados 1.439 artigos com os descritores ou palavras-chave relacionados à cárie, diagnóstico e inteligência artificial.

**RESULTADOS:**

Após análise, dezessete artigos científicos compuseram a amostra final. Desses artigos, 94,1% são bastante recentes, uma vez que foram publicados a partir de 2020. Apesar dos artigos terem sido desenvolvidos na perspectiva clínica, seus objetivos, resultados e conclusões sinalizam a possibilidade da efetividade da inteligência artificial como ferramenta estratégica para a epidemiologia. A utilização de fotografias frontais, laterais e oclusais permitiram o diagnóstico de cárie de todas as faces dos dentes.

**CONCLUSÃO:**

É fundamental investir-se em alternativas relacionadas à inteligência artificial e fotografias bucais para substituição dos tradicionais inquéritos epidemiológicos, o que permitirá o desenvolvimento pleno da vigilância em saúde bucal.

## INTRODUÇÃO

A cárie dentária configura-se como importante problema de saúde pública^1^, uma vez que sua prevalência média atinge 29% da população mundial^2^. Seu agravamento leva à dor, impactando significativamente na qualidade de vida e no absenteísmo no trabalho^3,4^. Apesar desse quadro, o poder público pode intervir na prevenção da cárie com medida coletiva eficaz e efetiva, como a fluoretação da água de abastecimento público^5^.

No âmbito do Sistema Único de Saúde (SUS) a abordagem à cárie ainda se caracteriza como enorme desafio especialmente pelo acúmulo de necessidades vivenciadas pela população, assim como pela organização do planejamento local com foco na equidade e com referencial epidemiológico^6^.

Os inquéritos epidemiológicos realizados no Brasil^7^permitem identificar a amplitude do problema no âmbito nacional, entretanto, não são capazes de identificar as necessidades de saúde bucal específicas de um determinado grupo populacional num dado território. Acresce que, quando o objetivo é identificar famílias e indivíduos em situação de risco na comunidade, há necessidade de aplicar os instrumentos de observação em todo o grupo populacional e não somente em uma amostra, o que torna inviável a realização de levantamentos epidemiológicos tradicionais para conhecer a situação de cárie dentária em âmbito local.

Em função disso, projeta-se a inteligência artificial como alternativa para a detecção de lesões de cárie, em especial a partir de imagens. Estudos têm sinalizado a utilização da radiografia odontológica^10,11^para a construção desse diagnóstico, o que evitaria a necessidade do exame clínico para essa finalidade. Entretanto, essa alternativa não é viável para finalidade epidemiológica considerando o seu alto custo para o sistema público de saúde e o próprio risco relacionado ao uso da radiação ionizante^12^.

Considerando essa dificuldade, estudos sinalizam a possibilidade de detecção de lesões de cárie a partir de imagens fotográficas associadas à inteligência artificial, as quais podem contribuir para a construção de referencial epidemiológico^13,14^, indispensável ao planejamento local em saúde bucal.

Com essas premissas, o objetivo do presente estudo foi realizar a síntese de evidências científicas relativas ao diagnóstico da cárie por meio de imagens fotográficas, mediadas pela inteligência artificial, que pudesse representar perspectivas da sua utilização na epidemiologia bucal.

## MÉTODOS

### Tipo de Estudo

Para realizar um mapeamento da produção científica da área da saúde que permitisse identificar os avanços e desafios relacionados ao diagnóstico da cárie por meio de imagens com utilização da inteligência artificial foi proposta a presente revisão de escopo^14^. Visando adequação à utilização desse tipo de revisão, foram seguidas as recomendações propostas pelo *Joanna Briggs Institute* ^15^ e os seus resultados foram organizados por meio do *Preferred Reporting Items for Systematic Review and Meta-Analyses Extension for Scoping Reviews* (Prisma-ScR)^16^.

Para a construção da pergunta inicial da pesquisa foi utilizada a estratégia PCC (População/Conceito/Contexto), na qual População = pessoas com cárie; Conceito = inquérito epidemiológico da cárie por imagem; e Contexto = uso da inteligência artificial. Com esses elementos foi possível a construção da seguinte pergunta norteadora da pesquisa: “Qual a situação atual dos inquéritos epidemiológicos da cárie desenvolvidos por meio de imagens com utilização da inteligência artificial?”.

Entretanto, a partir da estratégia de busca definida nas bases Biblioteca Virtual de Saúde (BVS), *Medical Literature Analysis and Retrievel System Online* (Medline), Web of Science (WoS) e *Abstract and Citation Database of Peer-Reviewed Literature* (Scopus), não foram identificados artigos que cumprissem os critérios previamente estabelecidos.

Em função dessa inadequação, foi reformulada a estratégia PCC passando o Conceito a ser definido como diagnóstico da cárie por imagem, mantendo-se a População (pessoas com cárie) e o Contexto (uso da inteligência artificial). A partir dessa alteração, a pergunta norteadora da pesquisa passou a ser a seguinte: “Qual a situação atual do diagnóstico da cárie desenvolvido por meio de imagens com utilização da inteligência artificial?”

### Fontes de Informação e Estratégia de Busca

As buscas foram realizadas de maio a agosto de 2024, tendo como data limite de publicação o dia 20 de agosto de 2024, nas bases de dados BVS, Medline, WoS e Scopus. A partir dos termos relacionados à pergunta da pesquisa sinalizados no *Medical Subject Headings* (MeSH), foram realizadas buscas de acordo com as especificidades de cada base de dados, cuja estratégia encontra-se detalhada no Quadro 1.

**Quadro 1:** Estratégia de busca utilizada nas bases de dados a partir dos descritores. Periódicos Capes, Brasil, 2024.

Base de dados	Estratégia de busca
BVS	*((artificial intelligence or machine learning or deep learning or fuzzy logic or computational intelligence or machine intelligence or computer reasoning or algoritm)) AND ((caries or lesion or dental cavity or dental cavities or white spot or decay)) AND ((diagnostic or diagnosis or detection or detecting or classification or classifying or screening or prediction or predicting))* Título, resumo, assunto
Medline	*(“artificial intelligence”[Title] OR “machine learning”[Title] OR “deep learning”[Title] OR “fuzzy logic”[Title] OR “computational intelligence”[Title] OR “machine intelligence”[Title] OR ((“computability”[All Fields] OR “computable”[All Fields] OR “computating”[All Fields] OR “computation”[All Fields] OR “computational”[All Fields] OR “computations”[All Fields] OR “compute”[All Fields] OR “computed”[All Fields] OR “computer s”[All Fields] OR “computers”[MeSH Terms] OR “computers”[All Fields] OR “computer”[All Fields] OR “computes”[All Fields] OR “computing”[All Fields] OR “computional”[All Fields]) AND “reasoning”[Title]) OR “algorithm”[Title] AND (“caries”[Title] OR “lesion”[Title] OR “dental cavity”[Title] OR “dental cavities”[Title] OR “white spot”[Title] OR “decay”[Title]) AND (“diagnostic”[Title] OR “diagnosis”[Title] OR “detection”[Title] OR “detecting”[Title] OR “classification”[Title] OR “classifying”[Title] OR “screening”[Title] OR “prediction”[Title] OR “predicting”[Title])*
WoS	*(artificial intelligence or machine learning or deep learning or fuzzy logic or computational intelligence or machine intelligence or computer reasoning or algoritm) AND (caries or lesion or dental cavity or dental cavities or white spot or decay) AND (diagnostic or diagnosis or detection or detecting or classification or classifying or screening or prediction or predicting) (Title)*
Scopus	*TITLE-ABS-KEY (artificial AND intelligence OR machine AND learning OR deep AND learning OR computational AND intelligence OR algorithm) AND TITLE-ABS-KEY (caries OR lesion OR dental AND cavity OR dental AND cavities) AND TITLE-ABS-KEY (diagnostic OR diagnosis OR detection OR detecting OR classification OR classifying OR screening OR prediction OR predicting)*

Capes: Coordenação de Aperfeiçoamento de Pessoal de Nível Superior; BVS: Biblioteca Virtual de Saúde; Medline: *Medical Literature Analysis and Retrievel System Online*; WoS: *Web of Science*; Scopus: *Abstract and Citation Database of Peer-Reviewed Literature*.

O fluxograma Prisma-ScR com o percurso investigativo relativo à identificação dos artigos, à definição dos critérios para seleção e elegibilidade e à avaliação e inclusão dos artigos encontra-se disponibilizado na Figura.

**Figura:** Fluxograma Prisma ScR.

BVS: Biblioteca Virtual de Saúde; Medline: *Medical Literature Analysis and Retrievel System Online;* WoS: *Web of Science;* Scopus: *Abstract and Citation Database of Peer-Reviewed Literature;* Prisma ScR: *Preferred Reporting Items for Systematic Review and Meta-Analyses Extension for Scoping Reviews.*

Vale ressaltar que a literatura cinzenta não foi consultada para preservar a validade, confiabilidade e consistência das análises da produção científica realizada em periódicos com revisão por pares.

### Coleta dos Dados

A partir de todas as informações dos artigos disponibilizadas em cada uma das bases, foi estruturado instrumento específico no software Microsoft Excel para tabulação dos dados e extração dos principais resultados.

Foram incluídos na revisão somente artigos completos publicados em inglês, sem delimitação de tempo, que continham os descritores ou palavras-chave relacionados à cárie, diagnóstico e inteligência artificial.

### Análise de Dados

A revisão dos artigos foi realizada de forma independente por dois revisores, cujas discordâncias foram solucionadas por consenso. Primeiramente, todos os títulos foram lidos com a intenção de identificar os estudos pertinentes para desenvolvimento da pesquisa. Nas situações nas quais a leitura do título não era suficiente para identificar a elegibilidade ou não do estudo, foi feita a leitura do resumo do artigo científico. As variáveis selecionadas para comporem o estudo relacionaram-se à autoria, ano, objetivos, tipo de estudo, população de estudo, fonte de dados, variáveis, resultados principais e conclusão.

Todos os artigos selecionados ao final da estratégia de busca foram exportados para o Mendeley, gerenciador de referências gratuito com a finalidade de organizar os documentos para posterior análise.

## RESULTADOS

A busca inicial nas bases de dados definida trouxe 1.439 artigos científicos, todos publicados em língua inglesa. Desses, 1.216 foram excluídos, pois não se alinhavam com o tema da pesquisa, e outros 135 artigos foram excluídos por estarem relacionados a imagens como radiografia, ultrassom, infravermelho, entre outras. Foram ainda excluídos editoriais, cartas e revisões (28), além de 14 artigos repetidos, uma vez que não respondiam diretamente ao foco da pesquisa. Dos 46 artigos restantes, foram excluídos 29 que não traziam os termos “photo”, ou “smartphone”, ou “artificial intelligence”, ou “deep learning” ou “machine learning”, elementos estratégicos para se abordar a possibilidade da articulação da epidemiologia com a inteligência artificial a partir de imagens. Ao final, 17 artigos compuseram a amostra para a presente revisão de escopo.

Ao serem analisados os 17 artigos selecionados, é possível observar que apenas um foi publicado antes do ano de 2020. A relação dos artigos com seu título, autor principal e ano de publicação encontra-se disponibilizada no Quadro 2.

**Quadro 2:** Título, autor principal e ano de publicação dos artigos incluídos na revisão de escopo, 2024.

Título do artigo	Autor/ano
*The accuracy of asynchronous tele-screening for detecting dental caries in patient-captured mobile photos: a pilot study*	Qari et al.^14^ (2023)
*Assessing a smartphone app (AICaries) that uses artificial intelligence to detect dental caries in children and provides interactive oral health education: protocol for a design and usability testing study*	Xiao et al.^17^ (2021)
*Automated caries detection with smartphone color photography using machine learning*	Duong et al.^18^ (2021)
*Deep learning application in dental caries detection using intraoral photos taken by smartphones*	Thanh et al.^19^ (2022)
*Mobile photographic screening for dental caries in children diagnostic performance compared to unaided visual dental examination*	Estai et al.^20^ (2021)
*Artificial Intelligence-powered smartphone application AlCaries improves at home dental caries screening in children: moderated and unmoderated usability test*	Al-Jallad et al.^21^ (2022)
*Detection of dental caries in oral photographs taken by mobile phones based on the YOLOv3 algorithm*	Ding et al.^22^ (2021)
*Development and evaluation of deep learning for screening dental caries from oral photographs*	Zhang et al.^23^ (2020)
*Visual diagnostics of dental caries through deep learning of non-standardized photographs using a hybrid yolo ensemble and transfer learning model*	Tareq et al.^24^ (2023)
*A computer-aided automated methodology for the detection and classification of occlusal caries from photographic color images*	Berdouses et al.^25^ (2015)
*Detecting white spot lesions on dental photography using deep learning: a pilot study*	Askar et al.^26^ (2021)
*A hybrid mask RCNN-based tool to localize dental cavities from real-time mixed photographic images*	Rashid et al.^27^ (2022)
*Caries detection on intraoral images using artificial intelligence*	Kühnisch et al.^28^ (2022)
*Caries detection with tooth surface segmentation on intraoral photographic images using deep learning*	Park et al.^29^ (2022)
*Simultaneous detection of dental caries and fissure sealant in intraoral photos by deep learning: a pilot study*	Xiong et al.^30^ (2024)
*Automated detection of posterior restorations in permanent teeth using artificial intelligence on intraoral photographs*	Engels et al.^31^ (2022)
*Employing CNN ensemble models in classifying dental caries using oral photographs*	AlSayyeda et al.^32^ (2023)

RCNN: *Region-Based Convolutional Neural Networks;* CNN: *Convolutional Neural Networks.*

O diagnóstico da cárie foi sinalizado por todos os artigos a partir da utilização da fotografia para simular a mesma imagem observada pelo cirurgião-dentista ao realizar um exame clínico. Sete estudos^14,17^ utilizaram smartphones para a tomada fotográfica da boca, três utilizaram câmera digital^23^ e outros sete estudos optaram por fazer a tomada fotográfica intraoral por meio de equipamentos fotográficos profissionais^26^.

Três estudos^14,17,21^tinham como objetivo métodos que permitiam a tomada fotográfica por pais ou familiares, sem a necessidade de um profissional de saúde para essa realização. Nessa mesma linha, um estudo^20^ propôs o desenvolvimento de um software para apoiar o cirurgião-dentista e os pacientes nas fases preliminares do diagnóstico. Todos os outros estudos estavam diretamente relacionados à possibilidade de o cirurgião-dentista detectar cárie dentária fora do ambiente clínico.

Quanto às unidades amostrais, houve grande variedade. Alguns estudos trabalharam com dentes extraídos, nos quais se utilizava a inteligência artificial para o diagnóstico da cárie a partir da imagem desses dentes. Desses, um estudo^24^ foi destinado a dentes anteriores, enquanto outros dois^18,25^ analisaram a face oclusal de pré-molares e molares, a mais atingida pela cárie dentária. Dois estudos^17,21^ tiveram a participação de pais e filhos na tomada fotográfica em ambiente domiciliar, com a finalidade de propiciar maior conhecimento e detecção precoce da cárie visando educação em saúde com os próprios membros da família. Um outro estudo^14^ promoveu a autofotografia por pacientes de clínica em curso de Odontologia, também com essa perspectiva educativa. A grande maioria dos estudos foi realizada com pacientes de clínicas de ensino ou de serviços de saúde. Quatro desses estudos foram realizados com pacientes de clínicas odontológicas ou hospitais, sendo que um estudo^28^ trabalhou com fotografias presentes no prontuário dos pacientes, enquanto três estudos foram feitos com fotos tomadas dos pacientes que se encontravam em tratamento^22,27,30^. Seis estudos foram realizados com pacientes de cursos de Odontologia, sendo que dois estudos^31,32^ investigaram fotos armazenadas nos registros dos cursos a partir de pesquisas conduzidas para fins educacionais e quatro trabalharam diretamente com os pacientes^19,23,26,29^. Destaca-se que um estudo^20^ foi desenvolvido com finalidade epidemiológica e teve como público-alvo escolares abrangendo crianças de 4 a 14 anos, cujos exames clínicos e fotografias foram realizadas na escola. Todas as imagens dos estudos selecionados para a presente revisão de escopo corresponderam a fotografias da boca, com especial destaque para os dentes.

Dois artigos^17,21^ não detalham o tipo de foto utilizada por se tratarem de estudos qualitativos que tinham como perspectiva obter o feedback sobre a usabilidade do aplicativo e de seu fluxo entre familiares, relacionado à estratégia de educação em saúde. Oito estudos utilizaram fotografias de dentes específicos^18,24^, assim como três estudos realizaram a tomada fotográfica de áreas específicas da arcada dentária^19,23,27^. De maior interesse para o presente estudo, quatro estudos realizaram tomadas oclusais. Desses estudos, um^30^ utilizou apenas a tomada oclusal, outro^22^ agregou fotografias frontais (face vestibular dos dentes anteriores) e dois^14,20^ incluíram, além das fotografias frontais, as fotografias laterais (face vestibular dos dentes posteriores).

Quanto ao tipo de estudo, dois estudos caracterizaram-se como estudos qualitativos^17,21^, ao passo que os demais foram estudos de acurácia. Desses, um estudo teve como foco a comparação entre o padrão de fotografias bucais tomadas por estudantes de Odontologia e pacientes^14^, enquanto outros quatro fizeram a comparação dos resultados do exame clínico com as fotografias^20,25,29,30^. Um estudo^27^ associou fotografias e radiografias para comparação dos resultados com o sistema de inteligência artificial previamente proposto. Nove preocuparam-se em comparar os resultados apresentados a partir da análise realizada apenas a partir das fotografias com o sistema de inteligência artificial^18,19,22^.

Um consolidado relativo aos aspectos abordados encontra-se disponibilizado na Tabela, enquanto as principais conclusões dos artigos analisados encontram-se disponibilizadas no Quadro 3.

**Tabela:** Total e percentagem das variáveis presentes na revisão de escopo, 2024.

Variável		Total	%
Período do estudo	Após 2020	16	94,1
	Até 2019	1	5,9
Equipamento para tomada fotográfica	Smartphones	7	41,2
	Câmera intraoral	7	41,2
	Câmera digital	3	17,6
Objetivo do estudo	Detectar cárie dentária em ambientes não clínico	13	76,5
	Analisar tomada fotográfica realizada por pais ou familiares	3	17,6
	Desenvolver software para apoiar nas fases preliminares do diagnóstico	1	5,9
Unidades amostrais	Pacientes de cursos de Odontologia	6	35,3
	Pacientes de clínicas ou hospital	4	23,5
	Dentes extraídos	3	17,6
	Pais e filhos em domicílio	2	11,8
	Crianças de 4 a 14 anos	1	5,9
	Autofotografia (pacientes de curso de Odontologia)	1	5,9
Região da boca fotografada	Dentes individualmente	8	47,1
	Tomadas fotográficas oclusais	4	23,5
	Regiões específicas da arcada dentária	3	17,6
	Sem identificação da região fotografada	2	11,8
Tipo de estudo	Qualitativo	2	11,8
	Acurácia	15	88,2

**Quadro 3:** Principal conclusão dos artigos incluídos na revisão de escopo de acordo com autor principal e ano de publicação, 2024.

Autor/ano	Conclusão
Qari et al.^14^ (2023)	A abordagem de teletriagem baseada na revisão de fotografias tiradas pelos participantes pode ser uma alternativa válida e confiável ao exame clínico.
Xiao et al.^17^ (2021)	Com o aplicativo AlCaries os pais podem usar smartphones comuns para tirar fotos dos dentes de seus filhos, detectar cárie da primeira infância, reduzir o risco de cárie e buscar tratamento em um estágio inicial da cárie.
Duong et al.^18^ (2021)	Aplicativo integrado de inteligência artificial para auxiliar as pessoas a darem um alerta precoce sobre suas condições de saúde bucal, assim como auxiliar os cirurgiões-dentistas a reduzir o tempo de exame clínico.
Thanh et al.^19^ (2022)	YOLOv3 e Faster RCNN são aplicações promissoras de inteligência artificial para detectar lesões de cáries cavitadas, entretanto, a acurácia e a sensibilidade dos quatro modelos na detecção de lesões de cáries iniciais permaneceram menores do que o esperado.
Estai et al.^20^ (2021)	A abordagem fotográfica do rastreio dentário produziu um nível diagnóstico aceitável de detecção de cárie, particularmente em crianças com dentição decídua.
Al-Jallad et al.^21^ (2022)	Os benefícios percebidos pelo uso do aplicativo AICaries incluem triagem conveniente de lesões de cáries em casa, informações sobre risco de lesões de cáries e educação, além do envolvimento dos familiares.
Ding et al.^22^ (2021)	Algoritmos de aprendizado profundo podem melhorar a distribuição desigual de recursos de saúde pública usando fotografias tiradas por smartphones para detecção especialmente de lesões de cáries primárias.
Zhang et al.^23^ (2020)	Algoritmos de aprendizado profundo podem melhorar a saúde pública ao permitir a triagem automática de doenças usando fotografias capturadas de câmeras fotográficas, podendo ser usado para autoexame.
Tareq et al.^24^ (2023)	O modelo pode melhorar o acesso à saúde bucal em áreas rurais com recursos limitados e tem o potencial de auxiliar em diagnósticos automatizados e aplicações avançadas de teleodontologia.
Berdouses et al.^25^ (2015)	Sistema de diagnóstico de cárie objetivo e totalmente automatizado para lesões de cárie oclusais com desempenho semelhante ou melhor que um cirurgião-dentista treinado.
Askar et al.^26^ (2021)	O aprendizado profundo mostrou precisão satisfatória na detecção de lesões de manchas brancas, principalmente fluorose.
Rashid et al.^27^ (2022)	A ferramenta desenvolvida apresentou-se eficaz para detectar regiões cariosas por meio de fotografias dentárias
Kühnisch et al.^28^ (2022)	Foi possível alcançar mais de 90% de concordância em detecção de cárie usando o método inteligência artificial com fotografias padronizadas de um único dente.
Park et al.^29^ (2022)	O aprendizado profundo com segmentação da superfície do dente é promissor para detecção de lesões de cáries em imagens fotográficas com câmera intraoral, com economia de tempo e custos.
Xiong et al.^30^ (2024)	O ToothNet alcançou detecção simultânea multitarefa em fotos intraorais, com vantagens na detecção de lesões de cáries e equivalência na detecção de selantes de fissuras, em comparação ao exame clínico feito pelo cirurgião-dentista.
Engels et al.^31^ (2022)	Foi possível categorizar diferentes tipos de restaurações posteriores em fotografias intraorais de forma automática e com boa acurácia.
AlSayyeda et al.^32^ (2023)	A estrutura de conjunto proposta para detecção de lesões de cáries é um passo em direção à construção de um sistema de suporte automatizado e eficaz.

RCNN: *Region-Based Convolutional Neural Networks.*

## DISCUSSÃO

Apesar do surgimento da inteligência artificial com potencial para intervir em processos na área da saúde datar de mais de setenta anos, suas limitações impediram sua ampla aceitação e aplicação no princípio, o que vai ocorrer a partir do advento do aprendizado profundo^33^. Conforme foi possível observar no presente estudo, apesar do primeiro dos artigos analisados ter sido publicado em 2015, 94,1% dos artigos analisados aconteceram a partir de 2020, o que permite afirmar que o interesse pela temática é bastante recente.

Os estudos preliminarmente levantados permitiram observar a grande produção científica relacionada ao diagnóstico clínico da cárie dentária mediado pela inteligência artificial, mas sempre tendo como referência a viabilidade dessas aplicações para a clínica odontológica, na lógica do atendimento individual. São relatados, nesses estudos, avanços a partir da inteligência artificial, em especial para predição (planejamento de cuidados odontológicos preventivos e cuidados de higiene bucal) e diagnóstico de cárie (pacientes com maior risco)^34,35^. Entretanto, a maioria desses estudos não foi elegível para desenvolvimento da presente revisão de escopo pelo interesse na aproximação com a área da epidemiologia.

Apesar de não terem sido identificados artigos que se referissem diretamente à questão da epidemiologia, vários estudos^18,20,22,31^ sinalizaram a necessidade de se propor pesquisas específicas que considerassem a inteligência artificial com esse foco. Tal sinalização vai ao encontro da necessidade de se identificar famílias e indivíduos de risco na comunidade.

Entre os objetivos dos estudos incluídos na presente revisão, alguns buscaram empoderar pais ou familiares para a detecção precoce da cárie e seu acompanhamento sistemático, colaborando sobremaneira com ações de educação em saúde^14,17,21,27^. Com isso, pretenderam contribuir para reduzir o risco de cárie e buscar tratamento em um estágio inicial da cárie^17^, o que seria essencial para o desenvolvimento efetivo da vigilância em saúde bucal.

Entretanto, quanto ao objetivo, destacaram-se artigos com a proposta de detectar a cárie dentária a partir da inteligência artificial por meio de imagens, fora do ambiente clínico. Diante dessas características, observou-se potencial contribuição para o uso dessas ferramentas em atividades voltadas à identificação de famílias e indivíduos de risco na área de abrangência de uma determinada unidade de atenção primária à saúde, a fim de reduzir a demanda espontânea e aumentar a demanda programada, sob os princípios da equidade.

Foram excluídos estudos que trabalharam com imagens como radiografias (periapicais, interproximais, panorâmicas), ultrassonografia, fluorescência ou infravermelho, as quais não seriam viáveis para se pensar na perspectiva da epidemiologia por seu custo e dificuldade de acesso.

Considerando essa limitação, a imagem eleita para responder aos objetivos do presente estudo foi a fotografia bucal. O registro por meio de fotografias no ensino odontológico é bastante comum, em especial com a finalidade de observação da situação previamente ao tratamento e após sua finalização.

Esse tipo de técnica utilizada na odontologia para registrar imagens detalhadas da boca do paciente permite que os profissionais de saúde bucal tenham uma visão clara e precisa das estruturas dentárias e tecidos moles, auxiliando no diagnóstico e no planejamento de tratamentos.

Entretanto, a utilização desse tipo de equipamento tornaria inviável a realização de diagnóstico de cárie por meio de imagens fotográficas considerando o custo do equipamento e os riscos relativos ao seu uso em determinados territórios. Dessa forma, uma alternativa para essa dificuldade seria a utilização de smartphones, presente em sete estudos ou câmeras digitais utilizadas em três estudos. Embora câmeras de smartphones possam não oferecer a mesma qualidade de imagem que seus homólogos profissionais ou intraorais, sua ampla disponibilidade, custo-benefício e aplicabilidade em pesquisas de saúde coletiva os tornam promissoras ferramentas com essa finalidade^35^. Além disso, os avanços sinalizados pela inteligência artificial demonstram capacidade na melhoria da qualidade da imagem fotográfica original por meio de tecnologia específica para esse fim^36^.

Outro elemento importante é o tipo de tomada fotográfica de acordo com a unidade amostral proposta pelo estudo. As fotografias de dentes específicos ou áreas de cada arcada dentária estiveram relacionadas a aspectos relacionados à precisão dos detalhes e testes a partir da inteligência artificial, o que não corresponde exatamente aos propósitos do presente estudo. Entretanto, seis artigos trazem contribuição fundamental para busca de solução para desenvolvimento de estudos epidemiológicos, pois trabalham com áreas da boca e o conjunto dos dados sinalizaram aproximação do diagnóstico da cárie. Pôde-se observar que estudos a partir de fotografias frontais e laterais permitiram o diagnóstico de cárie nas faces vestibulares dos dentes, ao passo que as oclusais ocuparam-se do diagnóstico da cárie nas faces de maior ocorrência da cárie dentária (oclusal e proximais)^37^.

Considerando os objetivos propostos pelos estudos, a maioria dos artigos caracterizou-se como estudos de acurácia, os quais permitem avaliar o quanto os resultados de uma aferição correspondem ao verdadeiro estado do fenômeno aferido^38^. A análise desses estudos permitiu observar que a maioria dos estudos sinalizou resultados promissores na utilização de fotografias bucais como substitutos do exame clínico realizado nos inquéritos epidemiológicos.

A conclusão de alguns estudos permitiu elementos que subsidiam estudos futuros visando a utilização da inteligência artificial em epidemiologia sinalizando que a avaliação fotográfica apresentou desempenho semelhante ou melhor do que o próprio exame realizado pelo cirurgião-dentista em levantamentos epidemiológicos^25,26,31,32^. Reforçando essa perspectiva, estudos sinalizaram resultados satisfatórios com sistemas criados tendo como referência a imagem fotográfica por meio da inteligência artificial^20,26,27,32^. Um artigo^20^ chama atenção para a possibilidade de tomadas fotográficas realizadas com precisão por pessoal auxiliar, o que seria muito importante para um maior envolvimento da equipe de saúde bucal nos inquéritos epidemiológicos. Da mesma forma, alguns artigos^14,17,21,27^ reportaram a tomada fotográfica realizada por pais, outros membros da família ou o próprio paciente, com resultados bastante satisfatórios.

Entre as conclusões, as que foram determinantes na contribuição dessa revisão de escopo para propor essas ferramentas como potenciais referem-se a artigos desenvolvidos a partir de objetivos relacionados à clínica, mas que contribuem significativamente com soluções para a saúde coletiva. Estudos sinalizaram a possibilidade de melhorar a distribuição desigual de recursos em saúde coletiva^22^, melhorar a saúde pública ao permitir a triagem automática de casos suspeitos de doenças^23^, melhorar o acesso à saúde bucal em áreas rurais^24^. Além disso, um estudo sinaliza a oportunidade de oferecer triagem potencialmente econômica e de fácil utilização^29^, proporcionando triagem de lesões de cáries dentárias confiável, acessível, menos invasiva, menos perigosa e menos demorada.

A análise dos resultados do presente estudo revela visão positiva sobre as possibilidades da Inteligência Artificial na área da saúde, coincidindo com estudo que destaca benefícios significativos para avanços no acesso dos pacientes aos serviços de saúde^39^.

Entretanto, apesar do avanço tecnológico e do valor agregado para processos de tomada de decisão em assistência à saúde proporcionados pela inteligência artificial, outros desafios continuam preocupando pacientes e os próprios profissionais de saúde. A utilização cuidadosa, ética e humanizada pelos profissionais de saúde desses recursos deve priorizar o respeito ao paciente, garantindo que a tecnologia seja uma aliada no cuidado. As implicações sociais e éticas, em especial relacionadas às medidas de privacidade e segurança para dados de pacientes e a real capacidade de monitoramento de saúde devem fazer parte desses cuidados^40^.

## CONSIDERAÇÕES FINAIS

É urgente o desenvolvimento de soluções baseadas nas evidências científicas e nos avanços tecnológicos que permitam aos profissionais de saúde, em especial os da atenção básica, desenvolverem o planejamento local em saúde bucal a partir de referencial epidemiológico.

É possível sinalizar a futura adoção de fotografias bucais como instrumentos estratégicos na substituição da complexa logística e da necessidade imperiosa do envolvimento do cirurgião-dentista na realização de inquéritos epidemiológicos, utilizados como referência única para o diagnóstico coletivo.

Também é importante observar que a acurácia obtida em vários estudos que utilizaram o diagnóstico de cárie mediado pela inteligência artificial a partir de fotografias, apesar de não ter o resultado ideal para a clínica, apresentam-se bastante satisfatórios para inquéritos epidemiológicos.

A partir das evidências sinalizadas na presente revisão, seria fundamental o desenvolvimento de estudos específicos que pudessem agregar à realidade epidemiológica brasileira os avanços da inteligência artificial.

E, uma vez que os avanços venham a ser alcançados, seria fundamental a inclusão desse tipo de informação e imagens no Prontuário Eletrônico do Cidadão do e-SUS APS^41^, viabilizando, com isso, a vigilância em saúde bucal.
